# Early brain–heart health manager–led multidimensional rehabilitation improves functional and psychosocial outcomes in hemorrhagic stroke: a retrospective cohort study

**DOI:** 10.3389/fneur.2025.1683579

**Published:** 2025-11-20

**Authors:** Yanzhen Shi, Liming Yan, Ming Yang

**Affiliations:** 1 Department of Cardiovascular Medicine, The Second People’s Hospital of Hunan Province (Brain Hospital of Hunan Province), Changsha, Hunan, China; 2 Department of Neurosurgery Cerebrovascular, The Second People’s Hospital of Hunan Province (Brain Hospital of Hunan Province), Changsha, Hunan, China; 3 Department of Emergency, The Second People’s Hospital of Hunan Province (Brain Hospital of Hunan Province), Changsha, Hunan, China

**Keywords:** hemorrhagic stroke, brain-heart health management, multidimensional rehabilitation, quality of life, post-stroke recovery

## Abstract

**Background:**

Hemorrhagic stroke is associated with substantial mortality and long-term disability. Conventional post-stroke care often overlooks the psychological and multidimensional needs of patients, limiting recovery potential. This study investigates the impact of an early, brain-heart health manager-led multidimensional rehabilitation program on functional, psychological, and quality-of-life outcomes in hemorrhagic stroke survivors.

**Methods:**

In this single-center, retrospective cohort study, we reviewed the medical records of 216 consecutive patients with spontaneous intracerebral hemorrhage admitted between January and December 2023. Patients were categorized into an intervention group (*n* = 108) or a control group (*n* = 108) according to the type of inpatient care they had received in routine clinical practice. The intervention group received standard care plus a structured rehabilitation program directed by certified brain-heart health managers, encompassing psychological support, individualized physical rehabilitation, nutritional counseling, and continuous follow-up for 3 months post-discharge. Primary outcomes included functional recovery assessed via the modified Rankin Scale (mRS). Secondary outcomes comprised emotional status (SAS, SDS), sleep quality (PSQI), fatigue severity (FSS), and health-related quality of life (SF-36). Change scores (*Δ* values) and multivariate logistic regression were used to analyze treatment effects and identify prognostic predictors. Patient grouping was based on pre-existing ward-level clinical management pathways rather than investigator allocation, ensuring fairness and minimizing selection bias.

**Results:**

Compared to the control group, the intervention group showed significantly greater improvements in mRS scores (mean 1.87 vs. 2.36, *p* < 0.001) and a higher proportion of favorable outcomes (mRS ≤ 2: 75.9% vs. 56.5%, *p* = 0.004). Marked reductions were observed in SAS, SDS, PSQI, and FSS scores, along with substantial gains across all SF-36 domains (all *p* < 0.001). Multivariate analysis identified early intervention, younger age, lower BMI, and lower baseline SAS/SDS scores as independent predictors of favorable prognosis.

**Conclusion:**

Early implementation of a multidimensional rehabilitation program led by brain-heart health managers significantly enhances neurological and psychosocial outcomes in hemorrhagic stroke patients. This integrated model offers a scalable strategy for optimizing recovery and quality of life in stroke care, while future multicenter prospective studies are warranted to validate and generalize these findings.

## 1 Introduction

Hemorrhagic stroke, characterized by spontaneous intracerebral hemorrhage in the absence of trauma, remains one of the most devastating subtypes of stroke, accounting for 20–40% of all cases globally (1). It is associated with significantly higher rates of early mortality, long-term disability, and reduced quality of life compared to ischemic stroke (2). Despite ongoing advances in acute medical management, the burden of post-stroke complications-ranging from physical impairments to psychological disturbances-continues to pose a major challenge to recovery and reintegration (3).

Conventional post-stroke care is often centered around stabilization, pharmacological therapy, and basic functional rehabilitation. However, this approach frequently neglects the multidimensional nature of stroke recovery, particularly in patients with hemorrhagic stroke, whose trajectories are complicated not only by neurological damage but also by emotional distress, fatigue, cognitive decline, and sleep disorders (4). Depression and anxiety are highly prevalent among stroke survivors and are strongly associated with poor adherence, delayed functional recovery, and diminished health-related quality of life (5). These limitations highlight an urgent need to reframe stroke rehabilitation through a more integrated, patient-centered, and interdisciplinary lens.

The brain-heart health management model represents an emerging paradigm in stroke care, combining evidence-based rehabilitation strategies with holistic support tailored to patients’ physical, psychological, and social needs. This model is led by certified brain-heart health managers-professionals with cross-disciplinary expertise in psychological counseling, nutrition, rehabilitation therapy, and health education. Through standardized training, these managers are equipped to deliver full-process, individualized care across all stages of recovery-from hospital admission to long-term follow-up (6). Unlike traditional nursing models, this approach emphasizes proactive engagement, continuous education, emotional regulation, and structured recovery planning-components that are often underutilized but essential for optimizing outcomes in stroke rehabilitation (7).

In recent years, China’s National Health Commission has recognized the value of this approach by launching a nationwide initiative to train brain-heart health managers for stroke prevention and recovery (8). While preliminary reports suggest potential benefits, there is a paucity of real-world data evaluating its effectiveness in hemorrhagic stroke patients-a group at particularly high risk for functional decline and psychological comorbidities. Moreover, few studies have investigated whether early implementation of this model, during the acute and subacute recovery phases, can result in meaningful improvements across multiple health domains (9, 10).

In this retrospective cohort study, we evaluated the impact of a brain-heart health manager-led, early multidimensional rehabilitation program on the recovery trajectories of patients with hemorrhagic stroke. We hypothesized that this integrative intervention-delivered in parallel with standard care-would be associated with superior outcomes in emotional well-being, sleep quality, fatigue severity, neurological function, and quality of life. Using validated instruments (2, 4, 8), including the Self-Rating Anxiety Scale (SAS), Self-Rating Depression Scale (SDS), Pittsburgh Sleep Quality Index (PSQI), Fatigue Severity Scale (FSS), the modified Rankin Scale (mRS), and the 36-item Short Form Health Survey (SF-36), we conducted a comprehensive evaluation of psychological, functional, and health-related quality of life outcomes 3 months post-discharge.

Our findings aim to address a critical gap in post-stroke care research: whether structured, holistic management led by specialized health professionals can translate into measurable gains in recovery and quality of life for individuals with hemorrhagic stroke. Given the growing global burden of stroke-related disability, this work offers important insights into scalable strategies for optimizing post-acute care and supporting functional independence in one of the most vulnerable patient populations.

## 2 Methods

### 2.1 Ethics statement

This study was reviewed and approved by the Ethics Committee of The Second People’s Hospital of Hunan Province (Hunan Provincial Brain Hospital; approval *No. HBH IEC_AF65_v1.1*). All study procedures were conducted in accordance with the ethical standards of the institutional and national research committees and with the 1964 Helsinki Declaration and its later amendments. The committee confirmed that the retrospective design, data collection process, and ward-level patient grouping used in this research met ethical requirements for human studies. Because this was a retrospective analysis, written informed consent was obtained from all participants or their legal guardians prior to data inclusion.

### 2.2 Study design and participants

This was a single-center, retrospective, observational cohort study conducted at The Second People’s Hospital of Hunan Province (Hunan Provincial Brain Hospital). We reviewed electronic medical records and follow-up data for all patients admitted between January 1, 2023, and December 31, 2023, with a primary diagnosis of spontaneous intracerebral hemorrhage.

Patients were categorized into two groups according to the type of inpatient rehabilitation care they had received as part of routine clinical practice: Intervention group – patients who received the brain-heart health manager–led multidimensional rehabilitation program in addition to standard care; Control group – patients who received standard post-stroke care alone. The rehabilitation program was implemented by certain clinical teams during the study period; patients were not randomized or prospectively allocated by the investigators. All inclusion and exclusion criteria were applied equally to both groups. Inclusion criteria: (1) diagnosis of spontaneous intracerebral hemorrhage according to the Chinese Guidelines for the Diagnosis and Treatment of Stroke (2020 edition) (5); (2) baseline modified Rankin Scale (mRS) score < 2 prior to stroke onset; (3) National Institutes of Health Stroke Scale (NIHSS) score ≥ 6 upon admission; and (4) aged between 40 and 80 years. Exclusion criteria: (1) intracranial infection, traumatic brain injury, or other space-occupying lesions; (2) pre-existing major psychiatric or neurodegenerative disorders; (3) inability to complete follow-up assessments due to severe cognitive impairment or language barriers. To reduce selection bias, only patients admitted during the same period, to the same department, and meeting identical criteria were included.

In this retrospective study, patient grouping reflected real-world clinical workflow rather than investigator-directed allocation. During the study period, the brain–heart health manager–led rehabilitation service was introduced in certain inpatient wards as part of a hospital-wide quality improvement initiative, while other wards continued to provide conventional care. Admission to a specific ward was determined by bed availability and clinical triage protocols, not by patient preference or physician discretion. This administrative allocation process minimized potential selection bias between groups. All inclusion and exclusion criteria were uniformly applied across wards, and no crossover occurred after enrollment.

To address the potential influence of primary treatment strategy on outcomes, the management approach for spontaneous intracerebral hemorrhage (surgical vs. conservative) was also recorded for each patient. Among all participants, 62 (28.7%) underwent surgical hematoma evacuation, while 154 (71.3%) received conservative medical management. The proportions of surgical patients were comparable between the intervention (*n* = 32, 29.6%) and control groups (*n* = 30, 27.8%; *χ*^2^ = 0.090, *p* = 0.764). This variable was incorporated as a covariate in multivariate logistic regression to minimize confounding and to isolate the independent effect of the rehabilitation intervention.

### 2.3 Intervention procedures

To ensure professional consistency and intervention fidelity, all brain–heart health managers participating in this study had completed the national certification program for brain–heart health managers organized by the National Health Commission of China. This standardized training includes core modules in neurological rehabilitation, cardiometabolic management, psychological counseling, and patient education, with a total duration exceeding 160 h of theoretical instruction and clinical practicum. Both Yanzhen Shi and Ming Yang are nationally certified brain–heart health managers, having successfully passed competency assessments and obtained formal qualification certificates. Their responsibilities encompassed individualized patient assessment, formulation of personalized care plans, delivery of structured psychological and rehabilitation interventions, and coordination of multidisciplinary follow-up. This certification process ensures the authenticity and reproducibility of the multidimensional rehabilitation protocol implemented in this study.

Patients in the control group received routine post-stroke care according to institutional standards. This included pharmacological treatment, vital sign monitoring, dietary recommendations, basic rehabilitation guidance, and general health education. Care was primarily reactive, with limited focus on psychological support, individualized rehabilitation planning, or structured follow-up after discharge.

In contrast, patients in the intervention group received the same standard care supplemented by a structured, full-process rehabilitation program led by certified brain-heart health managers. These professionals were registered nurses with over 5 years of clinical experience in stroke care who had completed formal training in psychological counseling, rehabilitation techniques, nutritional therapy, and patient education. The intervention began at hospital admission and extended through 3 months post-discharge. Upon enrollment, patients underwent a comprehensive baseline assessment addressing physical status, emotional well-being, social support, disease characteristics, and functional independence. A personalized care plan was formulated and recorded in a digital health file. Health education was delivered through a combination of printed materials, targeted bedside or small-group sessions, and family-involved discussions conducted by the brain–heart health managers. Weekly in-hospital group lectures were primarily attended by conscious, clinically stable patients in the subacute recovery stage, whereas for those with impaired consciousness or mobility, the content was conveyed to their family caregivers. This dual-audience format ensured continuity of education while maintaining clinical feasibility for the acute hemorrhagic stroke population. One-on-one bedside teaching and caregiver guidance were used to reinforce key topics such as secondary prevention, nutrition, and psychological support, tailored to each patient’s literacy level and clinical tolerance. Psychological support was systematically provided using evidence-based strategies such as cognitive reframing, guided meditation, progressive muscle relaxation, and music-assisted therapy to relieve anxiety and depressive symptoms. The brain–heart health managers collaborated closely with the hospital’s psychiatry and psychology departments. Cases presenting with persistent or severe mood symptoms (SAS or SDS ≥ 60) were referred to certified psychotherapists for specialized evaluation and cognitive–behavioral intervention following a predefined referral pathway. To ensure intervention fidelity, all psychological and rehabilitation activities followed a Standardized Operating Procedure (SOP) jointly developed by the departments of Nursing, Rehabilitation, and Psychiatry (see Supplementary Table S1). The SOP specifies frequency (≥3 sessions/week during hospitalization), duration (20–30 min), and core components for each evidence-based strategy. Exercise progression was individualized according to objective tolerance criteria, including Borg’s Rating of Perceived Exertion (RPE ≤ 13), stable vital signs, and absence of new neurological symptoms. Adjustments were documented in the electronic health record to maintain traceability and consistency. Early-stage functional rehabilitation was initiated as soon as patients were medically stable, with personalized exercise regimens adjusted according to tolerance and closely monitored to prevent fatigue or hemodynamic instability. Nutritional guidance was integrated throughout the inpatient stay and adapted to each patient’s comorbidities, body mass index, and laboratory parameters. Discharge planning involved detailed instructions on home-based rehabilitation, lifestyle modification, medication adherence, and warning signs of complications. After discharge, follow-up was maintained through regular phone calls, secure messaging applications (e.g., WeChat), and outpatient reviews, enabling continuous monitoring and timely intervention. This comprehensive, individualized, and process-driven approach was designed to address the multidimensional recovery needs of patients with hemorrhagic stroke, emphasizing both physical restoration and psychological resilience.

To ensure consistency and fidelity of intervention delivery, all participating brain–heart health managers completed the national certification program approved by the National Health Commission of China, which provides over 160 h of standardized training in neurological rehabilitation, cardiometabolic management, psychological counseling, and patient education. Both Yanzhen Shi and Ming Yang are nationally certified brain–heart health managers, having passed official competency assessments. Their certified training ensures the authenticity, professionalism, and reproducibility of the multidimensional rehabilitation protocol implemented in this study.

### 2.4 Outcome measures

Clinical outcomes were assessed at baseline and at the 3-month follow-up by evaluators blinded to group allocation. The primary outcome was functional recovery, defined by a favorable score (≤2) on the modified Rankin Scale (mRS), which evaluates post-stroke disability and independence (2). Secondary outcomes included psychological status, assessed using the SAS and SDS, with lower scores indicating better emotional well-being; sleep quality, measured by the PSQI, where higher scores denote poorer sleep; and fatigue severity, evaluated via the FSS, with scores ≥4 indicating clinically significant fatigue. Health-related quality of life was assessed using the SF-36, which covers eight domains: physical functioning, role limitations due to physical health, bodily pain, general health, vitality, social functioning, role limitations due to emotional problems, and mental health. To quantify treatment effects over time, change scores (*Δ* values) were calculated by subtracting baseline values from follow-up scores for each outcome variable. These multidimensional measures provided a comprehensive profile of each patient’s physical, psychological, and functional recovery following hemorrhagic stroke.

### 2.5 Statistical analysis

All statistical analyses were performed using SPSS version 22.0 (IBM Corp., Armonk, NY, USA). Continuous variables were expressed as means ± standard deviation (SD) and compared using Student’s t-tests for between-group comparisons and paired *t*-tests for within-group changes. Categorical variables were reported as counts and percentages, with group differences evaluated using the *χ*^2^ test. Multivariate logistic regression analysis was conducted to identify independent predictors of favorable outcomes (mRS ≤ 2). Variables with *p* < 0.05 in univariate analysis were entered into the multivariate model. Given the clinical importance of primary treatment strategy, “surgical status” (surgical hematoma evacuation *vs.* conservative management) was forcibly entered into the multivariate model as a mandatory covariate to control for potential confounding related to baseline treatment selection. Odds ratios (ORs) with 95% confidence intervals (CIs) were calculated. Baseline comparability was assessed before outcome analysis. To account for potential confounding, variables significant in univariate analysis were included in a multivariate logistic regression model. A two-sided *p*-value < 0.05 was considered statistically significant. Given the retrospective nature of this study and the limited sample size, propensity score matching (PSM) was not feasible. However, multivariate logistic regression incorporating major clinical and treatment-related covariates (including surgical status) was performed to minimize confounding and approximate the balancing effect achieved by PSM.

## 3 Results

### 3.1 Baseline characteristics

A total of 216 patients with hemorrhagic stroke were enrolled and evenly assigned to the intervention group (*n* = 108) and the control group (*n* = 108). Baseline characteristics—including age, sex, comorbidities, initial neurological severity, and multidimensional scale scores—did not differ significantly between the two groups (*p* > 0.05), demonstrating good comparability. Similarly, the proportion of patients who underwent surgical hematoma evacuation was comparable between cohorts (29.6% *vs.* 27.8%; *p* = 0.764), indicating balanced primary treatment strategies. Detailed baseline characteristics are presented in Table 1.

**Table 1:** Baseline characteristics of the two groups.

Variable	Control group (*n* = 108)	Intervention group (*n* = 108)	*t/χ^2^*	*p*-value
Age (years)	55.0 ± 4.3	54.8 ± 3.6	0.371	0.711
Primary treatment strategy (surgical)	30 (27.8%)	32 (29.6%)	0.090	0.764
BMI (kg/m^2^)	26.1 ± 1.8	26.3 ± 1.4	−0.779	0.624
SBP (mmHg)	147.7 ± 8.7	147.5 ± 9.3	0.162	0.871
DBP (mmHg)	87.7 ± 8.3	86.5 ± 7.8	1.095	0.275
Heart rate (bpm)	65.8 ± 6.4	66.4 ± 6.6	−0.678	0.498
Fasting glucose (mmol/L)	3.2 ± 0.6	3.1 ± 0.7	1.127	0.261
Baseline mRS	4.0 ± 1.1	3.9 ± 0.9	0.694	0.673
SAS score	53.1 ± 5.3	52.7 ± 6.0	0.528	0.598
SDS score	44.9 ± 4.1	44.6 ± 3.9	0.551	0.582
PSQI score	17.9 ± 1.0	18.1 ± 1.4	−0.192	0.848
FSS score	6.3 ± 0.2	6.4 ± 0.3	−0.786	0.433
SF-36 (Physical functioning)	47.7 ± 5.0	48.2 ± 4.5	−0.772	0.441
SF-36 (Role physical)	50.7 ± 4.3	50.5 ± 4.7	0.326	0.745
SF-36 (Bodily pain)	35.8 ± 3.8	35.7 ± 3.5	0.201	0.841
SF-36 (General health)	44.6 ± 3.9	44.2 ± 4.1	0.735	0.463
SF-36 (Vitality)	51.1 ± 4.7	51.3 ± 5.2	−0.297	0.767
SF-36 (Social functioning)	38.5 ± 4.4	38.6 ± 3.5	−0.185	0.854
SF-36 (Role emotional)	53.8 ± 4.9	53.6 ± 5.5	0.282	0.778
SF-36 (Mental health)	49.5 ± 5.4	49.8 ± 5.2	−0.416	0.678
Male	52 (48.1%)	56 (51.9%)	0.167	0.683
Diabetes	50 (46.3%)	50 (46.3%)	0.000	1.000
Hypertension	60 (55.6%)	55 (50.9%)	0.298	0.585
Hyperlipidemia	52 (48.1%)	47 (43.5%)	0.298	0.585
Smoking history	50 (46.3%)	48 (44.4%)	0.019	0.891
Alcohol history	52 (48.1%)	55 (50.9%)	0.074	0.785
Stroke side (Left)	34 (31.5%)	34 (31.5%)	0.000	1.000
Stroke type (Lobar hemorrhage)	36 (33.3%)	36 (33.3%)	0.000	1.000

### 3.2 Improvements in psychological status and sleep quality

**Figure 1:**
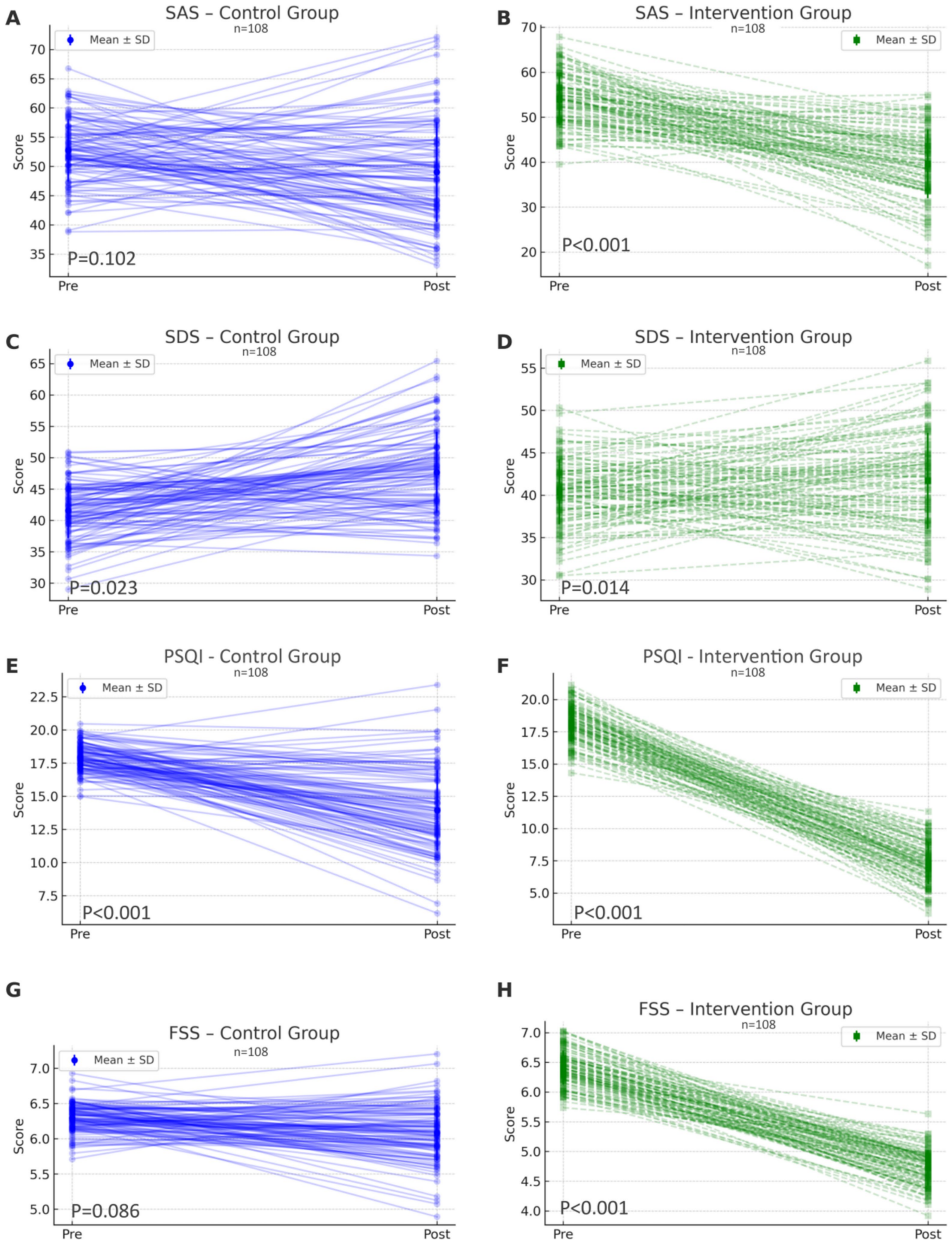
Improvements in psychological status, sleep quality, and fatigue severity before and after intervention in both groups (*n* = 108 per group). **(A,B)** Changes in Self-Rating Anxiety Scale (SAS) scores in the control group and intervention group, respectively. **(C,D)** Changes in Self-Rating Depression Scale (SDS) scores in the control group and intervention group. **(E,F)** Changes in Pittsburgh Sleep Quality Index (PSQI) scores in the control group and intervention group. **(G,H)** Changes in Fatigue Severity Scale (FSS) scores in the control group and intervention group. Each dot represents an individual patient’s score before and after the 3-month follow-up, connected by a line to demonstrate within-subject changes. Error bars indicate group mean ± standard deviation (SD). The intervention group exhibited greater improvements across all domains compared to the control group, highlighting the benefits of early multidimensional rehabilitation guided by a brain-heart health management model.

At the 3-month follow-up, patients in the intervention group exhibited significantly greater improvements in psychological well-being and sleep quality compared to the control group. Specifically, anxiety and depression levels, as measured by the SAS and SDS, were significantly reduced in the intervention group (SAS: 38.7 ± 7.6 vs. 48.2 ± 9.1; SDS: 40.5 ± 5.9 vs. 47.9 ± 6.8; *p* < 0.001 for both, Figures 1A–D). Similarly, improvements were observed in sleep quality and fatigue levels, with PSQI and FSS scores showing significantly better outcomes in the intervention group compared to controls (PSQI: 7.3 ± 1.8 vs. 13.5 ± 3.1; FSS: 4.7 ± 0.3 vs. 6.1 ± 0.4; *p* < 0.001 for both, Figures 1E–H). Paired within-group analysis confirmed that these improvements were statistically significant only in the intervention group. Collectively, these results suggest that the multidimensional early rehabilitation approach based on brain-heart health management effectively alleviated patients’ anxiety, depression, and sleep disturbances, while also reducing fatigue levels.

### 3.3 Functional recovery and neurological outcomes

After 3 months, the mRS score significantly improved in the intervention group compared to the control group (1.87 ± 0.62 vs. 2.36 ± 0.78, *p* < 0.001), suggesting enhanced neurological recovery. Notably, 75.9% of patients in the intervention group achieved favorable functional outcomes (mRS ≤ 2), compared to 56.5% in the control group (*χ*^2^ = 8.41, *p* = 0.004).

### 3.4 Quality of life enhancement across SF-36 domains

As shown in Table 2, patients in the intervention group exhibited significantly greater improvements across all eight domains of the SF-36 compared to the control group at the 3-month follow-up (all *p* < 0.001). Specifically, the intervention group outperformed the control group in physical functioning (53.3 ± 6.0 vs. 50.1 ± 4.5), role limitations due to physical problems (56.4 ± 4.6 vs. 51.9 ± 4.7), bodily pain (54.2 ± 5.9 vs. 47.3 ± 4.6), and general health perception (67.3 ± 5.5 vs. 52.7 ± 4.8). Notable improvements were also observed in vitality (65.2 ± 6.0 vs. 60.3 ± 6.1), social functioning (77.5 ± 6.2 vs. 65.1 ± 5.7), role limitations due to emotional problems (59.6 ± 7.0 vs. 53.6 ± 4.5), and mental health (57.6 ± 5.6 vs. 48.6 ± 5.4), with all differences reaching statistical significance (*p* < 0.001). These results indicate that early multidimensional rehabilitation based on a brain-heart health management model not only facilitates physical recovery but also leads to substantial improvements in emotional, social, and mental well-being. The comprehensive gains across all eight SF-36 domains highlight the potential of this integrative approach in enhancing post-stroke quality of life.

**Table 2:** Functional and quality of life outcomes after 3 months.

SF-36 domain	Control group (*n* = 108)	Intervention group (*n* = 108)	*t*	*p*-value
Physical functioning	50.1 ± 4.5	53.3 ± 6.0	−4.434	<0.001
Role physical	51.9 ± 4.7	56.4 ± 4.6	−7.111	<0.001
Bodily pain	47.3 ± 4.6	54.2 ± 5.9	−8.196	<0.001
General health	52.7 ± 4.8	67.3 ± 5.5	−13.667	<0.001
Vitality	60.3 ± 6.1	65.2 ± 6.0	−5.951	<0.001
Social Functioning	65.1 ± 5.7	77.5 ± 6.2	−6.728	<0.001
Role emotional	53.6 ± 4.5	59.6 ± 7.0	−7.493	<0.001
Mental health	48.6 ± 5.4	57.6 ± 5.6	−11.326	<0.001

### 3.5 Comparison of change scores (*Δ* values) between groups

**Figure 2:**
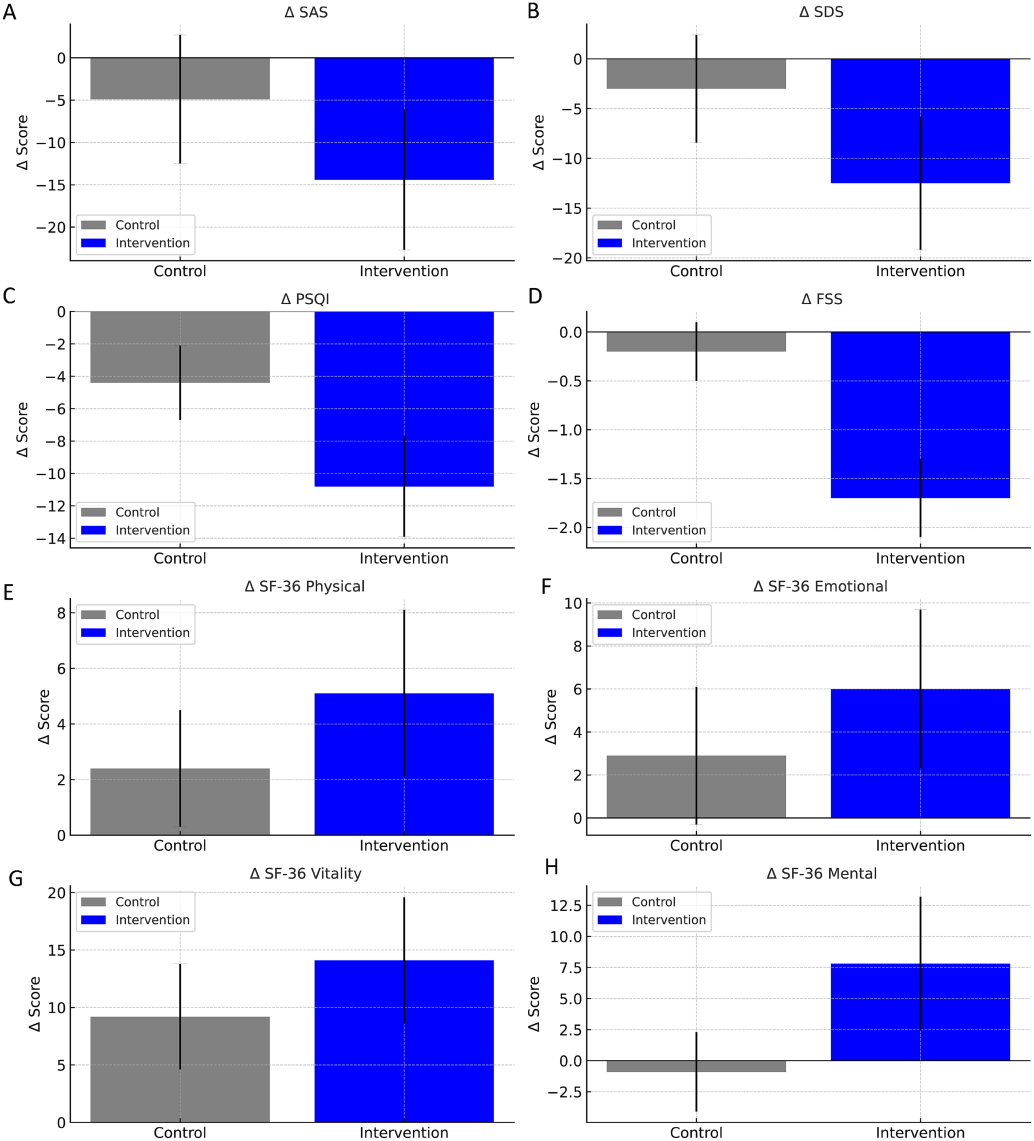
Between-group comparisons of mean change scores (*Δ* values) for psychological status, sleep quality, fatigue, and quality of life (*n* = 108 per group). Δ values were calculated as post-intervention minus pre-intervention scores. Bar graphs display the mean ± standard deviation (SD) for each group. Compared with the control group, the intervention group showed significantly greater improvements across all domains. **(A)** Self-Rating Anxiety Scale (SAS); **(B)** Self-Rating Depression Scale (SDS); **(C)** Pittsburgh Sleep Quality Index (PSQI); **(D)** Fatigue Severity Scale (FSS); **(E)** SF-36 Physical Functioning; **(F)** SF-36 Role Emotional; **(G)** SF-36 Vitality; **(H)** SF-36 Mental Health. The multidimensional early rehabilitation program based on brain-heart health management demonstrated superior efficacy in improving emotional well-being, sleep quality, fatigue, and health-related quality of life in patients with hemorrhagic stroke.

To quantitatively assess the efficacy of the early multidimensional rehabilitation intervention, we calculated the change scores (Δ values, defined as post-intervention minus pre-intervention) for each outcome measure (Figure 2). Compared to the control group, the intervention group demonstrated significantly greater improvements across all measures. Specifically, the reduction in SAS (*Δ* = −14.4 ± 8.3 vs. −4.9 ± 7.6) and SDS scores (Δ = −12.5 ± 6.7 vs. −3.0 ± 5.4) was more pronounced in the intervention group (*p* < 0.001 for both), indicating a substantial alleviation in anxiety and depressive symptoms. Similarly, for sleep quality and fatigue, the intervention group showed superior reductions in PSQI (*Δ* = −10.8 ± 3.1 vs. −4.4 ± 2.3) and FSS scores (*Δ* = −1.7 ± 0.4 vs. −0.2 ± 0.3), with both comparisons achieving statistical significance (*p* < 0.001), underscoring the benefits of the integrated management approach in mitigating sleep disturbance and fatigue severity. Improvements in quality of life were also significantly greater in the intervention group across all SF-36 domains. The most notable changes were observed in general health (*Δ* = +23.1 ± 6.0 vs. +8.1 ± 4.5), social functioning (*Δ* = +38.9 ± 6.4 vs. +26.6 ± 5.2), and mental health (*Δ* = +7.8 ± 5.4 vs. −0.9 ± 3.2), all with *p* < 0.001. These results suggest that the rehabilitation program not only enhanced physical recovery but also brought meaningful psychosocial and emotional benefits to post-stroke patients. Collectively, the between-group comparisons of Δ values provide robust evidence supporting the clinical advantage of early multidimensional intervention based on brain-heart health management in improving both psychological resilience and overall quality of life in patients with hemorrhagic stroke.

### 3.6 Multivariate logistic regression for prognostic predictors

To identify independent factors associated with favorable outcomes at 3 months following hemorrhagic stroke-defined as a mRS score ≤ 2-a multivariate logistic regression analysis was performed. Variables that showed statistical significance in univariate analysis (*p* < 0.05) were included in the multivariate model, with surgical status additionally incorporated as a mandatory covariate regardless of its univariate significance to ensure adjustment for baseline treatment strategy. As shown in Table 3, several baseline characteristics emerged as significant predictors of good prognosis. Being categorized into the intervention group was positively associated with favorable outcomes (OR = 1.177; 95% CI: 1.051–1.319; *p* = 0.005), suggesting that early multidimensional rehabilitation based on brain-heart health management significantly enhances recovery odds. Psychological status at baseline was also a strong predictor. Lower baseline scores on the SAS and SDS were both independently associated with better outcomes (SAS: OR = 0.821; 95% CI: 0.687–0.982; *p* = 0.030; SDS: OR = 0.854; 95% CI: 0.737–0.989; *p* = 0.035), highlighting the important role of emotional well-being in stroke recovery. Additional independent predictors included younger age (OR = 0.871; 95% CI: 0.765–0.991; *p* = 0.037), lower BMI (OR = 0.839; 95% CI: 0.713–0.987; *p* = 0.034), and a less severe baseline mRS score (OR = 0.855; 95% CI: 0.748–0.977; *p* = 0.021). These findings emphasize that initial clinical and psychological status are key determinants of post-stroke functional prognosis. Collectively, this multivariate model underscores that early intervention, along with favorable baseline physical and psychological profiles, significantly improves the likelihood of functional recovery in patients with hemorrhagic stroke. Notably, surgical status did not independently predict prognosis (*p* = 0.562), suggesting that the observed treatment benefit of the intervention was not confounded by differences in surgical management.

**Table 3:** Univariate and multivariate logistic regression for predictors of favorable prognosis (mRS ≤ 2).

Variable	*b*	S.E.	Wald *χ*^2^	Univariate OR (95% CI)	*p*-value (Univariate)	*b*^a^	S.E.^a^	Wald χ^2^^a^	Multivariate OR (95% CI)^a^	*p*-value (Multivariate)^a^
Intervention group	0.157	0.049	10.266	1.170 (1.063–1.288)	0.001	0.163	0.058	7.898	1.177 (1.051–1.319)	0.005
Baseline SAS score	−0.206	0.084	6.014	0.814 (0.690–0.959)	0.014	−0.197	0.091	4.687	0.821 (0.687–0.982)	0.030
Baseline SDS score	−1.142	0.385	8.799	0.319 (0.150–0.679)	0.003	−0.158	0.075	4.438	0.854 (0.737–0.989)	0.035
Age	−0.783	0.335	5.463	0.457 (0.237–0.881)	0.019	−0.138	0.066	4.372	0.871 (0.765–0.991)	0.037
Diabetes (yes vs. no)	−0.539	0.254	4.503	0.583 (0.355–0.960)	0.034	−0.105	0.098	1.148	0.900 (0.743–1.091)	0.900
BMI	−0.156	0.064	5.941	0.856 (0.755–0.970)	0.015	−0.176	0.083	4.496	0.839 (0.713–0.987)	0.034
Baseline mRS	−0.202	0.098	4.249	0.817 (0.674–0.990)	0.029	−0.157	0.068	5.331	0.855 (0.748–0.977)	0.021
Surgical status (surgical vs. conservative)	−0.092	0.178	0.265	0.912 (0.643–1.295)	0.607	−0.108	0.186	0.337	0.898 (0.626–1.288)	0.562
SBP	−0.094	0.245	0.147	0.910 (0.563–1.471)	0.701					
DBP	0.037	0.165	0.050	1.038 (0.751–1.434)	0.823					
Heart rate	0.147	0.385	0.146	1.158 (0.545–2.464)	0.703					
Fasting glucose	−0.183	0.285	0.412	0.833 (0.476–1.456)	0.521					
Baseline PSQI score	0.192	0.305	0.396	1.212 (0.666–2.203)	0.529					
Baseline FSS score	−0.064	0.250	0.066	0.938 (0.575–1.531)	0.798					
SF-36 Physical functioning	−0.015	0.062	0.228	0.985 (0.871–1.113)	0.633					
SF-36 Role physical	0.010	0.049	0.042	1.010 (0.917–1.112)	0.837					
SF-36 Bodily pain	0.012	0.046	0.069	1.012 (0.923–1.109)	0.792					
SF-36 General health	−0.018	0.056	0.104	0.982 (0.881–1.094)	0.747					
SF-36 Vitality	0.009	0.041	0.049	1.009 (0.930–1.095)	0.825					
SF-36 Social functioning	0.006	0.039	0.024	1.006 (0.930–1.089)	0.877					
SF-36 Role emotional	−0.008	0.048	0.030	0.992 (0.903–1.089)	0.863					
SF-36 Mental health	0.007	0.043	0.027	1.007 (0.925–1.097)	0.869					
Male sex	0.045	0.186	0.058	1.046 (0.729–1.501)	0.810					
Hyperlipidemia	−0.064	0.181	0.125	0.938 (0.658–1.336)	0.724					
Smoking history	0.030	0.178	0.029	1.030 (0.731–1.453)	0.865					
Alcohol history	0.042	0.175	0.058	1.043 (0.744–1.462)	0.810					
Stroke side (Left)	0.000	0.192	0.000	1.000 (0.692–1.445)	1.000					
Stroke type (Lobar hemorrhage)	0.000	0.191	0.000	1.000 (0.694–1.441)	1.000					

a Variables with *p* < 0.05 in univariate analysis that were included in multivariate logistic regression.

## 4 Discussion

This retrospective study provides compelling evidence supporting the clinical utility of a brain-heart health management model in the early rehabilitation of patients with hemorrhagic stroke. By integrating multidisciplinary, patient-centered care into standard post-stroke treatment, the model demonstrated superior outcomes across neurological, psychological, and quality-of-life dimensions. Specifically, patients who received individualized, process-driven interventions led by certified brain-heart health managers exhibited significantly better recovery profiles compared to those receiving conventional care alone. These findings highlight the potential of structured, holistic strategies in optimizing the complex recovery trajectory following hemorrhagic stroke.

The core proposition of this study is that stroke rehabilitation should extend beyond traditional paradigms, which largely focus on physical stabilization and delayed-phase functional training (11, 12). Conventional care often fails to address the intertwined psychological and social consequences of hemorrhagic stroke, which frequently include anxiety, depression, fatigue, and impaired sleep quality-each independently associated with poor long-term outcomes (13–15). In contrast, the proposed model emphasizes early intervention, continuity of care, and multidimensional support (16). Our results confirm the efficacy of this approach: patients in the intervention group showed significant reductions in anxiety (SAS) and depression (SDS), improved sleep quality (PSQI), reduced fatigue (FSS), and higher scores across all SF-36 domains of health-related quality of life. Moreover, 75.9% of patients in the intervention group achieved a favorable neurological outcome (mRS ≤ 2), compared to 56.5% in the control group.

This study advances current knowledge by addressing a critical gap in post-stroke care research-namely, the lack of integrated rehabilitation models tailored to hemorrhagic stroke survivors. While ischemic stroke dominates existing literature, hemorrhagic stroke presents greater rehabilitation challenges due to its more severe cerebral damage and higher comorbidity burden (17, 18). Our findings suggest that early engagement with an interdisciplinary care team-anchored by a brain-heart health manager-can mitigate these challenges and significantly improve patient trajectories. Multivariate logistic regression identified participation in the intervention group, lower baseline SAS and SDS scores, younger age, lower BMI, and better pre-stroke functional status as independent predictors of good recovery. To aid interpretation of the multivariate results, it should be noted that although the odds ratio (OR = 1.177) for the intervention group appears numerically modest, this value represents the adjusted independent effect after controlling for multiple baseline and clinical covariates—including age, BMI, baseline mRS, emotional status, and surgical status. In this context, an OR greater than 1.0 still indicates a statistically significant and clinically meaningful increase in the likelihood of achieving a favorable neurological outcome. The relatively small numerical magnitude reflects the statistical attenuation expected when multiple correlated predictors are entered into the same model rather than a weak clinical effect. Importantly, the observed OR aligns with effect sizes reported in comparable rehabilitation studies involving complex, multifactorial interventions, where incremental functional gains are often distributed across multiple domains rather than concentrated in a single metric. Taken together, these results suggest that the early multidimensional rehabilitation program contributed an independent, measurable, and clinically valuable improvement in patient prognosis despite adjustment for confounding factors. These results highlight the interplay between somatic and psychological health in shaping stroke outcomes and underscore the importance of early, comprehensive care.

Several limitations should be noted. First, the retrospective, non-randomized design may introduce selection bias, and causality cannot be established. Importantly, sensitivity analysis incorporating surgical status as a covariate confirmed that the independent association between the intervention and favorable outcomes remained robust, indicating that baseline treatment strategy did not materially bias the results. Although the baseline characteristics between the two groups were well balanced, PSM was not applied due to the limited sample size and retrospective data structure. Future studies employing PSM or inverse probability of treatment weighting (IPTW) would further strengthen the causal inference and reduce potential selection bias. Although baseline characteristics were comparable and multivariate analyses were performed, residual confounding from unmeasured variables is possible. Second, the study was conducted at a single centre, potentially limiting generalizability. Third, although validated instruments were used, outcomes such as fatigue and sleep quality relied on self-reported data and may be subject to response bias (19). Fourth, the follow-up period was limited to 3 months, and the long-term sustainability of the observed improvements remains to be established. Moreover, because this was a retrospective study, independent monitoring of intervention fidelity was not feasible. Although all procedures were implemented according to standardized SOPs, we acknowledge that the degree of adherence and protocol variation across providers could not be objectively verified, which may introduce implementation bias. Future multicentre, prospective studies with extended follow-up are needed to validate our findings and examine durability of effect. Alternative methodologies, such as randomized controlled trials, are essential to further strengthen the evidence base (20). Incorporating objective biomarkers, neuroimaging correlates, or digital health monitoring (e.g., actigraphy for sleep and fatigue) could enrich outcome assessments. Moreover, qualitative approaches-including semi-structured interviews and patient-reported experience measures-could offer deeper insight into patient satisfaction, adherence, and perceived benefits of the intervention. These complementary data sources would enhance understanding of how and why the model works and guide further refinement.

The implications of our findings extend beyond hemorrhagic stroke. The brain-heart health management model has broad applicability in the rehabilitation of patients with other complex neurological conditions, such as ischemic stroke, traumatic brain injury, and neurodegenerative disorders (11, 21, 22). As global health systems face increasing pressure from aging populations and chronic disease burdens, scalable, cross-disciplinary care models that integrate emotional, cognitive, and physical support will become increasingly critical (17, 23). The training and deployment of brain-heart health managers-professionals with expertise in nursing, rehabilitation, psychological counseling, and health education-offers a promising solution (24). This role addresses longstanding gaps in care continuity, patient engagement, and behavioral support across the healthcare continuum. Importantly, the qualifications and standardized training of the brain–heart health managers contributed to the reliability of intervention delivery and ensured that the multidimensional components were implemented according to national certification standards.

Looking forward, several key directions emerge. First, health systems should consider integrating brain-heart health management into standard stroke care pathways, particularly in high-burden, resource-constrained settings. Second, the development of national training programmes and certification standards for brain-heart health managers would ensure quality and consistency in service delivery. Third, future studies should evaluate the cost-effectiveness of this model and its impact on broader outcomes, including hospital readmissions, long-term institutionalization, and caregiver burden. Finally, leveraging telemedicine and digital health tools may enhance accessibility, adherence, and personalization of care, especially during post-discharge transitions.

**Figure 3:**
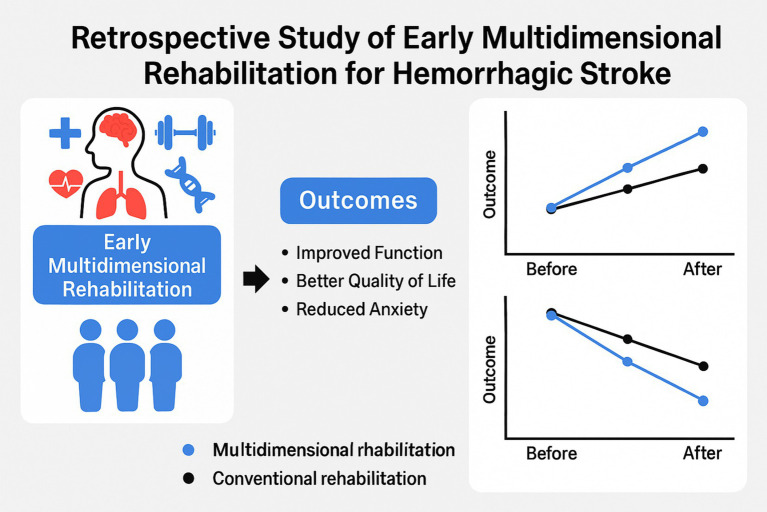
Graphical abstract summarizing the clinical impact of early multidimensional rehabilitation in hemorrhagic stroke.

In summary, this study demonstrates that early multidimensional rehabilitation guided by brain-heart health managers significantly improves functional, psychological, and quality-of-life outcomes in patients with hemorrhagic stroke (Figure 3). The approach addresses critical shortcomings in conventional care by emphasizing early engagement, personalized intervention, and integrated support. These findings provide a strong rationale for the adoption and further investigation of holistic, team-based care models to improve recovery in patients with complex neurological disorders. Future large-scale, multicenter randomized controlled trials are needed to confirm these benefits and assess the long-term sustainability and cost-effectiveness of the brain–heart health management model.

## 5 Conclusion

In conclusion, early brain–heart health manager–led multidimensional rehabilitation significantly improves functional and psychosocial outcomes in hemorrhagic stroke. This integrative model represents a practical and scalable innovation in post-stroke care. Future multicenter and prospective studies are warranted to further confirm its generalizability and long-term benefits.

## Data Availability

The original contributions presented in the study are included in the article/Supplementary material, further inquiries can be directed to the corresponding author.
