# Supplementary material for: Early brain–heart health manager–led multidimensional rehabilitation improves functional and psychosocial outcomes in hemorrhagic stroke: a retrospective cohort study
Source: Front Neurol. 2025 Nov 20;16:1683579. doi: 10.3389/fneur.2025.1683579 (PMC12675196; doi:10.3389/fneur.2025.1683579)
Supplement: Supplementary file 1 [file Data_Sheet_1.docx]

**Supplementary Table S1.** Standardized Operating Procedure for Psychological Support and Rehabilitation Progression

This Standardized Operating Procedure (SOP) provides detailed guidance for the implementation of the multidimensional rehabilitation program led by certified Brain–Heart Health Managers in patients with hemorrhagic stroke. It aims to ensure consistency, reproducibility, and intervention fidelity across all cases.

1. Purpose and Scope

To standardize the delivery of psychological support, physical rehabilitation, and multidisciplinary collaboration for hemorrhagic stroke patients receiving the Brain–Heart Health Manager–led rehabilitation program. This SOP applies to all certified Brain–Heart Health Managers, rehabilitation nurses, physiotherapists, and collaborating psychologists involved in patient care.

2. Professional Roles and Responsibilities

| Role | Main Responsibilities |
| --- | --- |
| Brain–Heart Health Manager | Conduct baseline assessments, design individualized rehabilitation plans, deliver psychological support and health education, monitor progress, and coordinate multidisciplinary care. |
| Psychotherapist/Psychiatrist | Provide specialized evaluation and therapy for patients with severe or persistent emotional disorders (SAS or SDS ≥ 60); advise on complex psychological interventions. |
| Rehabilitation Therapist | Guide exercise training, assess tolerance using Borg RPE scale, monitor vital signs, and prevent complications such as fatigue or hemodynamic instability. |
| Nutritionist | Develop and adjust nutritional plans based on comorbidities, BMI, and biochemical indicators. |
| Family Caregiver | Participate in bedside teaching, home-based rehabilitation, and psychological support reinforcement. |

3. Workflow Overview

Step 1: Initial Assessment – Conduct comprehensive evaluation covering neurological status, emotional state (SAS/SDS), sleep (PSQI), fatigue (FSS), physical function (mRS, SF-36), and social support.

Step 2: Individualized Plan Development – Based on assessment findings, establish personalized rehabilitation goals and document them in the electronic health record.

Step 3: Intervention Implementation – Deliver standardized sessions according to defined frequency, duration, and content (see Section 4).

Step 4: Monitoring and Adjustment – Reassess tolerance using Borg RPE, vital signs, and patient feedback; adjust intensity accordingly.

Step 5: Referral Protocol – Refer to psychiatry/psychology when mood scores (SAS or SDS ≥ 60) or clinical judgment indicates need for specialized care.

Step 6: Discharge and Follow-up – Provide home rehabilitation guidance, maintain weekly phone/WeChat follow-up for 3 months, and record adherence and adverse events.

4. Standardized Intervention Components

A. Psychological Support

• Frequency: ≥ 3 sessions per week during hospitalization.
• Duration: 20–30 minutes per session.
• Environment: Quiet, well-lit ward area with patient comfort ensured.
• Core methods: Cognitive reframing, guided meditation, progressive muscle relaxation, and music-assisted therapy.
• Tools: Validated scripts, relaxation audio, and visual cue cards.
• Documentation: Each session logged with date, duration, techniques used, and patient response.

B. Exercise Progression

• Initiation: Begin once patient achieves hemodynamic stability (SBP < 160 mmHg, HR < 100 bpm, no new neurological symptoms).
• Frequency: Daily sessions (20–40 minutes), guided by rehabilitation therapist.
• Intensity: Adjust using Borg Rating of Perceived Exertion (target ≤ 13).
• Safety monitoring: Discontinue if dizziness, excessive fatigue, or abnormal vital signs occur.
• Progression: Gradual increase in activity complexity (from passive range-of-motion to active-assisted and task-oriented training).

C. Nutritional Counseling

• Initial evaluation: BMI, fasting glucose, lipid profile, renal and hepatic function.
• Diet plan: Energy intake 25–30 kcal/kg/day; protein 1.0–1.2 g/kg/day; sodium < 5 g/day.
• Monitoring: Adjust based on laboratory results and appetite changes.

D. Family and Caregiver Education

• Mode: Bedside instruction and printed educational leaflets.
• Content: Emotional care techniques, dietary guidance, warning signs of complications, and adherence reinforcement.
• Goal: Enable caregivers to act as active participants in long-term recovery.

5. Documentation and Quality Control

All intervention activities must be recorded in the electronic rehabilitation log. Weekly peer supervision is conducted by the chief Brain–Heart Health Manager to ensure consistency and adherence. Deviations from the SOP must be justified and reported to the multidisciplinary review committee.

6. Criteria for Referral and Escalation

• SAS or SDS score ≥ 60, or patient-reported severe emotional distress.
• Suicidal ideation, psychosis, or unmanageable behavioral symptoms.
• Poor adherence despite repeated motivational support.
• Complex multimorbidity requiring specialist input.

7. Objective Criteria for Personalization and Adjustment

• Borg RPE scale ≤ 13.
• Stable vital signs: SBP < 160 mmHg, DBP < 100 mmHg, HR < 100 bpm.
• SAS/SDS improvement ≥ 10% from baseline before increasing session intensity.
• PSQI or FSS scores used to adjust rest-exercise balance.

8. Follow-Up and Evaluation

After discharge, follow-up occurs weekly via telephone or WeChat for 3 months. Health managers document psychological status, sleep quality, and adherence. If deterioration is observed, in-person reassessment is arranged.

9. References and Regulatory Basis

This SOP is based on national standards for Brain–Heart Health Manager training (National Health Commission of China, 2022) and institutional stroke rehabilitation protocols approved by The Second People’s Hospital of Hunan Province.
